# Secondary reproduction in the herbaceous monocarp *Lobelia inflata*: time-constrained primary reproduction does not result in increased deferral of reproductive effort

**DOI:** 10.1186/1472-6785-14-15

**Published:** 2014-05-20

**Authors:** Patrick William Hughes, Andrew M Simons

**Affiliations:** 1 Department of Biology, Carleton University, Ottawa, Canada K1S 5B6

**Keywords:** Life-history evolution, Semelparity, Iteroparity, Facultative iteroparity, *Lobelia inflata*

## Abstract

**Background:**

Although semelparity is a life history characterized by a single reproductive episode within a single reproductive season, some semelparous organisms facultatively express a second bout of reproduction, either in a subsequent season (“facultative iteroparity”) or later within the same season as the primary bout (“secondary reproduction”). Secondary reproduction has been explained as the adaptive deferral of reproductive potential under circumstances in which some fraction of reproductive success would otherwise have been lost (due, for example, to inopportune timing). This deferral hypothesis predicts a positive relationship between constraints on primary reproduction and expression of secondary reproduction. The herbaceous monocarp *Lobelia inflata* has been observed occasionally to express a secondary reproductive episode in the field. However, it is unknown whether secondary reproduction is an example of adaptive reproductive deferral, or is more parsimoniously explained as the vestigial expression of iteroparity after a recent transition to semelparity. Here, we experimentally manipulate effective season length in each of three years to test whether secondary reproduction is a form of adaptive plasticity consistent with the deferral hypothesis.

**Results:**

Our results were found to be inconsistent with the adaptive deferral explanation: first, plants whose primary reproduction was time-constrained exhibited decreased (not increased) allocation to subsequent secondary reproduction, a result that was consistent across all three years; second, secondary offspring—although viable in the laboratory—would not have the opportunity for expression under field conditions, and would thus not contribute to reproductive success.

**Conclusions:**

Although alternative adaptive explanations for secondary reproduction cannot be precluded, we conclude that the characteristics of secondary reproduction found in *L. inflata* are consistent with predictions of incomplete or transitional evolution to annual semelparity.

## Background

Semelparity (in plants, “monocarpy”) has generally been studied from a demographic perspective that directly contrasts semelparity and iteroparity. Cole [1] found that the fitness advantage gained by an iteroparous over a semelparous strategy was very slight; a semelparous organism had to produce only a single additional offspring to offset the advantage of surviving to reproduce again. This finding was initially puzzling because it implied that semelparity should be common. Later authors noted that differential juvenile-to-adult mortality, age-specific schedules of reproduction and other life-history traits affect the fitness of the semelparous habit [2-9]. More recently, iteroparity and semelparity have also been thought to be endpoints of a continuum of life histories [10-21].

There is mounting empirical support for this continuum hypothesis; semelparous species display significant reproductive variation, and many reproductive modes are not “pure” or “classical” semelparous or iteroparous habits. Some semelparous organisms facultatively reproduce a second time (i.e. in a discrete bout that is discontinuous from the first). For organisms subject to seasonal variation in living conditions, this second bout can occur either: (1) within the same season as the first (primary) bout, where it has been loosely termed “secondary reproduction” or “partial semelparity” [11,15,21-24]; or (2) in a subsequent season, where it has been termed “facultative iteroparity”, or “facultative polycarpy” in plants [5,23,25,26]. To avoid confusion between these terms, in this paper we refer exclusively to “secondary reproduction”, operationally defined as a second, non-continuous reproductive episode in an organism considered to be semelparous. This is meant to encompass both within-season “secondary reproduction” and true “facultative iteroparity”. We also refer to an occurrence of secondary reproduction or facultative iteroparity as a “secondary reproductive episode” or SRE. Here, we investigate the life-history significance of SREs in *Lobelia inflata*.

That a semelparous organism may be facultatively capable of reproducing more than once is surprising because the general benefit of semelparity is theorized to be the gain of a demographic advantage through a simultaneous, exhaustive reproductive episode [2,6,7,10,17,27-29]. Identifying the conditions under which secondary reproduction or facultative iteroparity may occur is correspondingly important.

The most common explanation for SREs is that they are an adaptively plastic response to environmental stressors that constrain primary reproduction. Under conditions where normal semelparous reproduction may result in lost reproductive effort, deferral of reproductive effort to a second reproductive bout can realize fitness gains [11,19,22,23,30,31]. We term this the “adaptive deferral hypothesis”. According to this explanation, since the SRE is elicited as an adaptively plastic response to constrained reproduction, semelparous organisms capable of SREs should display ‘pure’ semelparity only under ideal (unconstrained) conditions; constraints on primary reproduction should result in deferral of reproductive effort to a second bout. An important corollary to this explanation is that the likelihood that reproductive effort will be deferred to a SRE (as well as the degree to which total reproductive effort is deferred) is proportional to the severity of the restriction of primary reproduction. Therefore, according to the adaptive deferral hypothesis, SREs should: (1) be expressed at more opportune times than primary reproductive episodes, since deferral of reproductive effort to an equally or more greatly constrained time would not increase fitness; and (2) show a proportional relationship between the degree to which reproductive effort is deferred to an SRE and the degree of constraint on the primary reproductive episode.

Several studies have supported the ability of the adaptive deferral explanation to account for SRE display. For example, when primary reproduction was artificially restricted in the crab spider *Lysiteles coronatus*, mothers compensated by reducing reproductive effort invested in the primary reproductive episode, and deferred reproductive effort to a second [11]. There is also evidence for a positive relationship between the degree of reproductive constraint and the expression of secondary reproduction - in a study of the semelparous Eresid spider *Stegodyphus lineatus*, Schneider *et al.* also artificially restricted primary reproduction [19], but did so at different times – either at two or ten days after hatching. In these studies, both of which were performed in the lab, the authors found that the likelihood of observing a SRE was proportional to the severity of restriction of primary reproduction. In the field, the latent capacity to facultatively express an SRE has been observed in the erpobdellid leeches *Erpobdella octoculata*[32] and *Nephelopsis obscura*[33,34].

The most common alternative explanation for secondary reproduction is that semelparous organisms with a recent evolutionary transition from iteroparity to semelparity, or with a highly plastic expression of parity, may display reproduction a second time when the fitness cost of doing so is not high. We term this alternative explanation the “transitional hypothesis”, since it emphasizes the continuity between iteroparity and semelparity. According to this view, SREs may occur as part of a presumably adaptive (but potentially vestigial or maladaptive) opportunistic reaction norm: if selection for semelparity is not strong, an SRE can realize additional fitness. This hypothesis implies that, for seasonal monocarps, the expected value of an SRE should correlate with the likelihood of being able to successfully complete that SRE. As semelparity is considered to be an adaptation to low adult survival [7,17,35], where conditions are not conducive to prolonged adult survival (i.e. late reproduction may risk loss of reproductive potential) selection for more complete or extreme semelparity will be favoured over life-histories where adults survive to exhibit SREs. For semelparous organisms in seasonal environments, the timing of reproduction is usually critical, and while plants reproducing early may prolong the reproductive bout in time without losing fitness, the importance of semelparity increases as reproduction is initiated later in the season, since these plants will then under stronger selection for rapid, simultaneous and exhaustive reproduction (because they will have no future opportunity to realize fitness). The transitional hypothesis therefore predicts: (1) that SREs may occur at any time, including inopportune times; and (2) that increased SRE display will be associated with earlier primary reproduction.

Our focus for this study is the semelparous herb *Lobelia inflata* (Campanulaceae), which, according to several lines of evidence, evolved semelparity comparatively recently*.* Semelparity is considered a derived trait elsewhere in the genus *Lobelia*: of 21 species of *Lobelia* L. sect. *Lobelia*, 20 are iteroparous, with only *L. inflata* being consistently reported as semelparous. *L. appendiculata* has also been variably described as monocarpic [36]; even so, this character is clearly derived from a polycarpic ancestor [37]. Furthermore, *Lobelia cardinalis*, the species most closely related to *L. inflata*, is iteroparous [38], and previous studies have demonstrated the lability of parity in the genus *Lobelia*[8].

Fitness in semelparous herbs like *L. inflata* depends on a schedule of reproduction that exhausts energy reserves just as the season ends; selection on reaction norms governing reproductive strategies is strong and reproducing at the right time is critical [39]. For many monocarps, the developmental transition to reproduction is initiated in response to and depends on the plants’ evolved response to seasonal cues [29,40-51]. For *L. inflata*, transition from a prereproductive vegetative phase to a reproductive phase—marked by the formation of a reproductive stalk and termed “bolting”—occurs in response to seasonal changes in day length and light quality [39,40,52-59]. The timing of initiation of reproduction is critical, since plants need to exhaust accumulated resources before the season ends; plant phenology and offspring phenotype are especially sensitive to the timing of initiation of reproduction [48]. Due to this sensitivity, an effective experimental method to restrict the ability for plants to complete primary reproduction without damaging plant tissue is to manipulate photoperiod to manipulate the cue for initiation of reproduction. We note that restricting primary reproduction might also have been accomplished via flower or inflorescence destruction, but plants generally do not provide maternal care to seeds, and many plants exhibit a compensatory growth response to herbivory or flower destruction that would confound the response to the restriction of reproduction itself, an issue that is avoided by using time restriction to constrain primary reproduction [31,60-63].

Laboratory experimental studies have an advantage over observational (or correlational) approaches when it is important to attribute response to a particular agent, or when a question demands extending the observed natural phenotypic range of expression [64]. For *L. inflata*, an observational study *in situ* cannot be used to assess the extent to which reproductive effort is deferred, because adverse conditions in the field suppress the expression of SREs. We therefore used a lab-based phenotypic manipulation experiment to study the underlying potential (unexpressed in the field) for plants to express an SRE, and thus whether, and to what extent, plants exhausted their reproductive potential before then onset of winter. For this experiment, we subjected replicate groups of plants in a growth chamber to increasingly severe time constraints on primary reproduction by manipulating the photoperiod and light intensity to mimic the natural changing conditions of the site of collection (Petawawa, ON). An increase in the expression of SREs in response to more severe time constraints during the primary reproductive episode would be consistent with the “adaptive deferral hypothesis” comparable to SREs elicited by restricted primary reproduction in other taxa [19,24]. This hypothesis specifically predicts that SRE expression should be proportional to restriction severity; i.e. the less time a plant has to complete reproduction, the more likely it is to display a SRE, and the greater investment it will make in the SRE. Alternatively, if SREs in *L. inflata* are a vestigial expression of the iteroparous history of the genus *Lobelia* or an adaptation to an ancestral environment, time-restricting primary reproduction should be negatively correlated with SRE expression if SREs waste reproductive potential (especially under temporal constraints that select for more extreme semelparity), or should be uncorrelated if SREs have little or no fitness cost. It should be noted that, even if results show that SREs are not effective as a deferral of reproductive effort, alternative explanations to the vestigial hypothesis exist. For example we cannot discount the possibility that SREs may have been adaptive in ancestral environments (or in areas with winters even milder than the southern margins of its current range), or that SREs are adaptive for unknown reasons.

## Results

### Likelihood of SRE occurring

**Figure 1:**
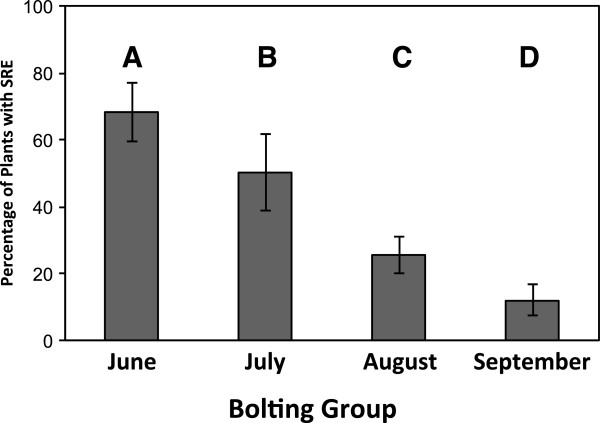
**Proportion of plants expressing a Secondary reproductive episode (SRE) by bolting group (BG).** The four bolting groups were experimentally produced using a photoperiod manipulation. Levels not sharing letters indicate significantly different means by Tukey HSD test following a factorial ANCOVA performed for illustrative purposes.

According to our binomial logistic regression model, the timing of bolting was a strong predictor of likelihood of SRE expression (χ^2^_6_ = 274.52, *p* < 0.005; Table 1). The likelihood of SRE expression was higher in early-bolting, less time-constrained plants, and lower in late-bolting, more time-constrained plants (Figure 1). Likelihood of SRE display did not depend on year or prebolting rosette size (Table 1). A second binomial regression—run as a mixed-model analysis with genotype included as an additional random effect—did not explain significantly more variation in the response (χ^2^_1_ = 1.87, *p* = 0.171), and thus genotypic lineage did not predict likelihood of SRE expression.

**Table 1:** Results of binomial logistic regression analysis of factors affecting likelihood of SRE expression

**Term**	**Parameter estimate (±SE)**	**Wald chi-square statistic**	**df**	**p**	**Estimated odds ratio**
Constant	−2.34 (0.35)	45.25	1	0.23	-
Year = 2008	−0.30 (0.20)	2.27	1	0.74	1.07
Year = 2009	0.07 (0.17)	0.16	1	1.07	0.74
Year = 2010	0	-	-	-	1.00
Bolting month = June	2.93 (0.24)	149.82	1	< 0.001*	18.79
Bolting month = July	2.45 (0.23)	108.93	1	<0.001*	11.61
Bolting month = August	0.81 (0.24)	11.49	1	<0.001*	2.26
Bolting month = September	0	-	-	-	1.00
Size	0.02 (0.01)	3.33	1	0.07	1.02

The four bolting groups were experimentally produced using a photoperiod manipulation, and this manipulation was repeated for three years from 2008-10. Prebolting plant size was measured as the length (in mm) of the longest living leaf of the unbolted rosette. The regression model significantly predicts likelihood of SRE expression (χ^2^ = 274.52, df = 6, p < 0.005). Odds ratios express relative likelihood of SRE expression.

*p < 0.05.

### Proportion of total fruits allocated to SRE

**Figure 2:**
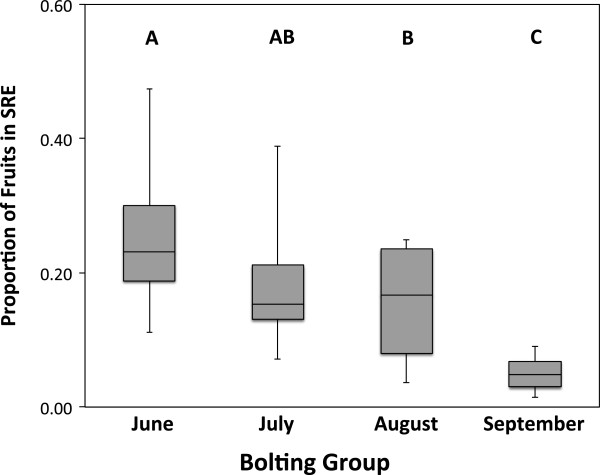
**Proportion of fruit allocated to SRE across the four experimental bolting groups.** Boxes indicate 2nd/3rd data quartiles, horizontal lines show median value and whiskers show range of data dispersal. Levels not sharing letters indicate significantly different means by Tukey HSD test following a factorial ANCOVA performed for illustrative purposes.

According to a second binomial logistic regression model, the timing of bolting was a strong predictor of the proportion of total fruit expressed during the SRE (χ^2^_32_ = 2200.57, *p* < 0.005; Table 2). This analysis showed that early-bolting, less time-constrained plants expressed a greater proportion of their total fruits in the SRE than did late-bolting, more time-constrained plants (Figure 2). Year and prebolting rosette size were not significant predictors of the proportion of total fruits produced during the SRE (Table 2). This binomial logistic regression analysis was also repeated with genotype included as an additional random effect, but again the resulting mixed model did not explain significantly more variation in the response (χ^2^_1_ = 2.34, *p* = 0.126), and therefore genotypic lineage did not predict the proportion of total fruit expressed in the SRE.

**Table 2:** Results of binomial logistic regression analysis of factors affecting proportion of total fruits allocated to SRE

**Term**	**Parameter estimate (±SE)**	**Wald chi-square statistic**	**df**	**p**	**Estimated odds ratio**
Constant	−4.88 (0.20)	589.81	1	<0.001*	-
Year = 2008	−0.40 (0.32)	1.51	1	0.22	0.67
Year = 2009	0.18 (0.23)	0.61	1	0.44	1.19
Year = 2010	0	-	-	-	1.00
Bolting month = June	3.35 (0.17)	389.09	1	<0.001*	28.50
Bolting month = July	2.96 (0.17)	300.49	1	<0.001*	19.30
Bolting month = August	1.71 (0.18)	87.45	1	<0.001*	5.52
Bolting month = September	0	-	-	-	1.00
Size	0.02 (0.00)	0.37	1	0.54	1.01

The four bolting groups were experimentally produced using a photoperiod manipulation, and this manipulation was repeated for three years from 2008-10. Prebolting plant size was measured as the length (in mm) of the longest living leaf of the unbolted rosette. The regression model is a significant predictor of the response (χ^2^ = 2200.57, df = 32, p < 0.005). Odds ratios express relative likelihood of fruit being allocated to the SRE.

*p < 0.05.

### Fruit number

A factorial ANCOVA model including bolting month, prebolting rosette size and year, significantly predicted the mean total number of fruits produced per plant (*F*_*12,956*_ = 36.53, p < 0.01; Additional file 1: Table S1). Tukey HSD tests on bolting group revealed two homogenous subsets; September-bolting plants produced the most fruit (mean = 46.93, SE = 2.9), which was significantly more than any other group, with July-bolting plants (mean = 36.0, SE = 1.1), August-bolting plants (mean = 33.4, SE = 1.0), and June-bolting plants (mean = 31.2, SE = 2.1) all producing significantly fewer fruit. This model indicated that bolting month, year, bolting month*year and prebolting rosette size (the covariate) were all significant predictors of the response.

### Timing of SRE

**Figure 3:**
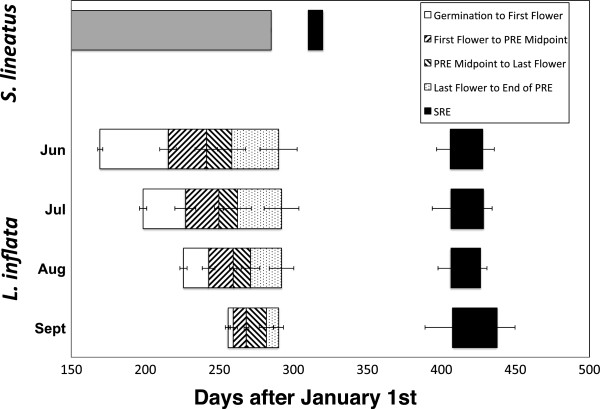
**Reproductive phase diagram comparing *Lobelia inflata *bolting groups (means from 2008-10) and *Stegodyphus lineatus *(data from Schneider and Lubin 1997).** Boxes indicate successive stages of growth and whiskers indicate standard error (some data unavailable for *S. lineatus*).

SRE expression occurred from January to early February the following year. A large proportion of plants (431 of 969, or 41.7%) expressed a SRE, all between 95 and 127 days after the termination of primary reproduction and apparent senescence (Figure 3). The overall mean time between the cessation of primary reproduction and the initiation of the SRE was 108.3 days (SE = 16.6 days). Because early- and late-bolting plants ended primary reproduction at approximately the same time (~October 15), SREs for all BGs took place at approximately the same time: late January to mid February of the year following primary reproduction.

### Seed germination

Our analysis revealed no phenotypic differences among offspring resulting from primary reproduction and SREs with respect to germination fraction (*F* = 0.069, df = 1,101, *p* = 0.794) or days to germination (*F* = 1.63, df = 1,101, *p* = 0.204; Additional file 2: Table S2).

## Discussion

The adaptive deferral hypothesis, which predicted that plants under the severest time constraints would be the most likely to express SREs, was not supported by our findings. Each of the four measures we assessed in this manipulation study—likelihood of SRE expression, proportional reproductive investment in SRE, total fruits produced, and timing of reproduction—showed a trend opposite to what would be expected given that the deferral hypothesis were true, and instead were consistent with the transitional hypothesis.

First, we observed that late-bolting, heavily time-constrained plants were less likely than early-bolting, less time-constrained plants to express a SRE (Figure 1), and produced proportionately fewer SRE fruits if they did (Figure 2). This pattern is inconsistent with the deferral hypothesis, which predicts that time-restriction of primary reproduction should be positively correlated with both SRE expression and investment. Second, although SREs were given the opportunity for expression in the lab, they have no opportunity for expression under field conditions in a temperate climate: in our growth-chamber study, reproduction generally ended in October, followed by an inter-reproductive period of over 100 days. Also, plants transplanted from our field site to a glasshouse in October 2008 (just before the first snow) exhibited SREs the following January (P.W. Hughes pers. obs.). It is therefore clear that deferral of reproductive effort to an SRE does not contribute to reproductive success, contrary to predictions of the adaptive deferral hypothesis. In examples where SREs are explained by adaptive deferral of reproductive effort, the SRE generally takes place either: (1) shortly after primary reproduction (i.e. within 25 days for *S. lineatus*; Figure 3); or (2) in the next season, as for the pitcher plant *Wyeomyia smithii*[65]. In either case, SREs increase fitness because SRE occur under conditions that are as or more favourable than the conditions under which primary reproduction took place [19,24]. Although in principle it is possible that the inter-reproductive period for *L. inflata* would be longer under field conditions than it was in the lab, and an SRE could occur the following spring, to the best of our knowledge this has not yet been observed. For these reasons, we conclude that SRE expression in *L. inflata* cannot be accounted for by the adaptive deferral hypothesis.

Our results did support the transitional hypothesis, both because SREs were produced at an inopportune time, and because the underlying allocation pattern showed that heavily time-constrained plants were less likely to defer offspring than were less time-constrained plants. If these underlying trends are also expressed in the field, these trends suggest that: (1) early-bolting plants that opt to defer reproduction may die with significant unspent reproductive potential, representing a substantial fitness cost; and (2) late-bolting plants compensate for time-constraints by producing flowers more quickly as the season progresses. Furthermore, the observation that no differences in seed viability were detected between primary and secondary reproductive episodes indicates that SREs cannot be discounted as simply pseudo reproduction that incurs no energetic costs; this further suggests that any reproductive effort allocated to SREs in the field is wasted. For these reasons, our main conclusion is that SREs do not represent an effective avenue for the adaptive deferral of reproductive effort in *L. inflata*, which is the primary hypothesis explaining why SREs exist in semelparous organisms; that deferral results in wasted reproductive effort may have contributed to the evolution of semelparity in *L. inflata*.

It is important to recognize the limits on the interpretation of data collected under growth-chamber conditions, but also that we are not making assumptions about how SRE would be expressed in the field. It is because we expect the expression of SRE to be suppressed under field conditions that we used phenotypic manipulation to expose the trait of interest across a particular array of laboratory conditions. Here, we use photoperiod, a cue known to be ecologically relevant and that elicits a strong reproductive response in *L. inflata*[66] to reveal an otherwise unobservable expression of SRE. We thus make an assumption common to all phenotypic manipulations: that the expression of a trait (SRE) will not respond in an opposite manner to a manipulation (photoperiod) in the lab compared to its potential expression in the field. We focused on photoperiod cues in *L. inflata*; other cues, correlated with an unknown factor germane to expectation of realized reproductive effort, may influence the developmental decision to defer resources for a SRE. Finally, we note that our study was designed to test the adaptive deferral hypothesis, and rejection of this hypothesis does not conclusively establish that SREs are maladaptive or nonadaptive in *L. inflata*. Further study of SRE expression in *L. inflata* elsewhere in its range would allow us to better assess the fitness consequences for plants expressing SREs.

## Conclusions

In conclusion, we found that while *L. inflata* plants had the ability to produce viable seeds in SRE fruits, the pattern in which they did so was inconsistent with the adaptively plastic deferral of reproductive effort seen in animal systems: here, time-constraining primary reproduction resulted in semelparity with reduced secondary reproduction. This norm of reaction is consistent with the expression of vestigial iteroparity that decreases as time constraints increase. Where seasonality is strong and winter is long (i.e. throughout most of *L. inflata*’s current range), SREs likely represent a loss of reproductive effort. Thus, secondary reproductive episodes in *L. inflata* appear to be a vestige of their iteroparous evolutionary past.

## Methods

### Test species – *Lobelia inflata* (Campanulaceae)

*L. inflata* is an herbaceous plant in the family Campanulaceae, and is found throughout Eastern North America. It prefers sandy soils and thrives on the margins of roadways or in disturbed areas. Like many monocarpic plants, *L. inflata* has two discrete phases of life: vegetative (accumulating resources as it is a growing rosette) and reproductive (expending resources on the production of offspring). Once a plant forms a stalk (‘bolts’), vegetative growth ceases and leaves senesce. Reproduction occurs acropetally (i.e. in series from basal to apical positions) as the stalk grows, with most plants producing between 10-100 fruit, followed by senescence [66]. To trigger bolting, thresholds for rosette size, light intensity and photoperiod (day length) must be met [39]. The “decision” to bolt is irreversible; however, if rosettes do not bolt in a given year, they are capable of overwintering [59,67,68]. At Petawawa, *L. inflata* rosettes typically bolt any time from late May to mid July during their second season post germination.

Reproduction occurs along the stalk (or small branches) as flowers are produced acropetally over the course of the reproductive season. Because the timing of the decision to bolt indicates an irreversible transition to the terminal reproductive phase of a plant’s life history, and late bolting limits the time available to a plant to survive and reproduce [39], bolting is a critical fitness trait. After primary reproduction finishes, plants normally senesce and become dry, brittle and brown. In the field, secondary reproduction, which is very rare, is observed most often in warm spells in late autumn. Although the main stalk is apparently “dead” or fully senesced, SREs occur through the production of new shoots from leaf axils. Typically, shoots produced during a SRE do not grow taller or longer than 10 cm, remain unbranched, and flower acropetally until resources are exhausted.

*L. inflata* is an obligately self-fertilizing hermaphrodite, and produces offspring that are genetically identical to their parent. A closed tube of fused anthers ensures self-fertilization; pollen is released directly onto the stigma of the same flower. Aside from enforcing self-fertilization, the anther tube also prevents outcrossing by acting as a mechanical barrier to pollen release. No examples of outcrossing have been recorded, and heterozygosity in the Petawawa population appears to be zero [69].

### Seed collection and rosette growth

*L. inflata* seeds were collected in October 2007 from the Petawawa Research Forest in Ontario, Canada (Lat. 45°99’N, Long. 77°30’W). Because it is self-fertilizing, *L. inflata* persists in isolated genotypic lineages; thus, to reduce the likelihood of studying an atypical genotype, seeds from spatially separated parental plants (a minimum of 50 m apart) were used to found an experimental population of 21 (potential) genetic lineages. Eight of these 21 lineages were used in the 2008 experiment, and all 21 were used in the 2009 and 2010 experiments. Note that although we include genotype as a factor in analyses, a study aimed at assessing genetic variation for expression of SRE would comprise more than 8-21 genotypes; our intent here was to include a representative random sample of existing genetic variation.

### Manipulation of effective reproductive season length

We manipulated available time to reproduce by controlling the timing of rosette bolting (initiation of reproduction) in a two-stage design implemented yearly from 2008-2010. In the vegetative, or first stage, we grew plants from seed to bolting rosette under ideal conditions: 400-800 seeds of each genotype were germinated on moist filter paper in a BioChambers SG-30 seed germinator under a regimen of 12 h /12 h (day/night) at 20°C with constant 85% humidity for 10-14 days. Seedlings were then planted in sterilized soil in 32-well cell soil trays and transferred to a Biochambers AC-40 growth chamber (on a 24°C/18°C 16 h/8 h day/night regimen) for approximately 40 days of growth. After this growth period, bolted plants were transferred to the reproductive chamber for the second, reproductive, stage. In each year, this process was repeated four times; once each to produce bolted rosettes by June 15th, July 15th, August 15th and September 15th. This created four bolting groups – one per month from June to September. Plants that did not bolt on the 15th (+/- 1 day) were not included in the bolting group. Each bolting group (BG) was translocated (on the 15th of the month) into another AC-40 growth chamber designed to simulate the outdoor environment at Petawawa (following outdoor photoperiod and light intensity via astronometric clock; temperature 20°C/16°C day/night). We followed the BGs as they expressed primary reproduction, senesced, and expressed SREs (or not). Plants were monitored until no further flowering occurred.

We observed each plant and recorded: (1) initial (prebolting rosette) size; (2) whether or not plants expressed a SRE; (3) how many fruits were produced during both primary reproduction and SRE; and (4) what proportion of fruits was allocated to the SRE. Initial plant size was assessed as rosette size, measured as the length of longest living leaf (LLL), the best available surrogate measure of biomass in *L. inflata*[66,67]. Two traits were followed to assess secondary reproduction: we noted whether a plant produced a SRE, defined as any flowering occurring after initial senescence of the main reproductive stalk. Second, we counted the number of fruits produced in both the primary and secondary reproductive episodes. The proportion of all fruits expressed in the SRE was used as the estimate of the reproductive effort invested in the SRE. After harvest we measured the stalk height of all plants and subjected a subsample of seeds (from only the June and September BGs) to test viability.

### Seed viability

We examined whether SREs produced viable seeds to determine if reproductive effort realized during a SRE resulted in potential fitness gains. To assess viability, we measured seed length and germination fraction of subsamples of fruits from both the primary and secondary reproductive episodes from all plants in the June and September BGs in all three years. Seed measurements were calculated from digital photos (after allowing 72 hours for water absorption) and a stage micrometer. Germination fraction was assessed by placing seeds on moistened filter paper on Petri plates in an SG-30 seed germinator running on a 24°C/14°C 16 h/8 h 85% humidity schedule for 45 days, examining seeds every other day under an Olympus B061 light microscope.

### Statistical analyses

Three statistical approaches were used to evaluate plant investment in secondary reproduction, and for two of these a GLMM (mixed model) was run to assess the importance of random effects as well as fixed effects. First, to examine the likelihood of SRE expression, we used binomial logistic regression to model the equation governing SRE likelihood and to compare the relative weighting of bolting month, year, genotype, and prebolting rosette size on the response. A binomial logistical regression was chosen since the response was binary (i.e. “SRE expressed/No SRE expressed”), and can be understood as a series of Bernoulli trials with the log of the odds ratio as the linking function. Second, to examine the count of SRE fruits as a proportion of total fruits produced, we again used binomial logistic regression (where we modeled the total number of fruits as the set of trials and the number of fruit produced during the SRE as the number of “events” or “successes” in that set) to model the equation governing SRE investment and compare the relative weighting of different predictors on the response. Finally, to predict total (primary and secondary) fruit produced, we used a factorial ANCOVA. This analysis was run to examine whether or not SRE expression was associated with a direct cost (i.e. having fewer total fruit). Before running this ANCOVA, we used a Shapiro-Wilk test to assess the distribution of all predictor and response variables: none had a distribution that significantly differed from normality. For the ANCOVA analysis, we treated bolting group and year as fixed effects and included prebolting rosette size as a covariate.

To examine whether genotypic lineage was a significant predictor of SRE expression, we ran all three analyses as mixed-effect models (i.e. GLMMs), including genotype as a random effect. To estimate total variance attributable to genotype, we used REML-based estimation [70,71]. We then compared the fixed-effect-only models to the mixed-effect models using a likelihood ratio test; where the model including the random effect did not explain significantly greater proportion of total variation in the response, we discarded it and used a fixed-effect binary logistic regression or ANCOVA instead. Tests showing a significant effect of bolting group were followed by Tukey HSD post-hoc tests to assess which bolting groups differed. Seed viability proportions were compared by F-test. All statistical analyses were performed in SPSS 21.0 (IBM Corp).

## Availability of supporting data

The data set supporting the results of this article is available in the Data Dryad repository, doi:10.5061/dryad.m02bt, http://URL: https://datadryad.org/resource/doi:10.5061/dryad.m02bt[72].

## Competing interests

The authors declare that they have no competing interests.

## Authors’ contributions

PWH conducted the manipulation experiment, collected all experimental data, performed statistical analysis and contributed to the drafting and writing of this manuscript. AMS conceived of this study, performed statistical analysis and contributed to the drafting and writing of this manuscript. Both authors read and approved the final manuscript.

## Supplementary Material

Additional file 1: Table S1 Results of factorial ANCOVA of factors affecting mean number of total fruits produced per plant. The covariate included in the model was prebolting rosette size (measured by the length of the longest leaf), and the two main effects were bolting month (June, July, August or September) and year (2008, 2009, or 2010). R^2^ = 0.314, adjusted R^2^ = 0.306. Click here for file

Additional file 2: Table S2 Seed viability data for fruits from plants of June and September bolting groups. Data shown is calculated using data from all years (2008-2010). No significant differences between seeds produced in primary or secondary reproductive episodes were found for mean days to germination or germination fraction. Click here for file
