# Supplementary material for: Secondary reproduction in the herbaceous monocarp Lobelia inflata: time-constrained primary reproduction does not result in increased deferral of reproductive effort
Source: BMC Ecol. 2014 May 20;14:15. doi: 10.1186/1472-6785-14-15 (PMC4030501; doi:10.1186/1472-6785-14-15)
Supplement: Additional file 1: Table S1 — Results of factorial ANCOVA of factors affecting mean number of total fruits produced per plant. The covariate included in the model was prebolting rosette size (measured by the length of the longest leaf), and the two main effects were bolting month (June, July, August or September) and year (2008, 2009, or 2010). R2 = 0.314, adjusted R2 = 0.306. [file 1472-6785-14-15-S1.docx]

**Table S1 – Results of factorial ANCOVA of factors affecting mean number of total fruits produced per plant. The covariate included in the model was prebolting rosette size (measured by the length of the longest leaf), and the two main effects were bolting month (June, July, August or September) and year (2008, 2009, or 2010). R^2^ = 0.314, adjusted R^2^ = 0.306.**

| Source | df | F | *p* |
| --- | --- | --- | --- |
| Corrected Model | 12 | 36.53 | <0.001* |
| Intercept | 1 | 58.68 | <0.001* |
| Size | 1 | 132.63 | <0.001* |
| Bolting month | 3 | 43.15 | <0.001* |
| Year | 2 | 42.56 | <0.001* |
| Bolting month * Year | 6 | 10.99 | <0.001* |
| Error | 956 |  |  |
| Total | 969 |  |  |
| Corrected Total | 968 |  |  |
