# Supplementary material for: Secondary reproduction in the herbaceous monocarp Lobelia inflata: time-constrained primary reproduction does not result in increased deferral of reproductive effort
Source: BMC Ecol. 2014 May 20;14:15. doi: 10.1186/1472-6785-14-15 (PMC4030501; doi:10.1186/1472-6785-14-15)
Supplement: Additional file 2: Table S2 — Seed viability data for fruits from plants of June and September bolting groups. Data shown is calculated using data from all years (2008-2010). No significant differences between seeds produced in primary or secondary reproductive episodes were found for mean days to germination or germination fraction. [file 1472-6785-14-15-S2.docx]

**Table S2 - Seed viability data for fruits from plants of June and September bolting groups. Data shown is calculated using data from all years (2008-2010). No significant differences between seeds produced in primary or secondary reproductive episodes were found for mean days to germination or germination fraction.**

| Bolting Month | Reproductive Episode | *n* | Mean Days to Germination (±SE) | Mean  Germination Fraction (±SE) |
| --- | --- | --- | --- | --- |
| June | Primary | 343 | 21.69  (2.82) | 0.474  (0.043) |
|  | Secondary | 1487 | 21.56  (1.45) | 0.413  (0.029) |
| September | Primary | 418 | 20.54  (1.18) | 0.466  (0.070) |
|  | Secondary | 1469 | 23.66  (1.27) | 0.438  (0.028) |
